# Dynamic Localization of Endoplasmic Reticulum during Tetrahymena Conjugation

**DOI:** 10.17912/micropub.biology.001300

**Published:** 2024-09-30

**Authors:** Sabrice Guerrier, Michael Patterson, Kaitlin Crofton, Michael Tucker, Shyhiem Walker

**Affiliations:** 1 Department of Biology, Rollins College, Winter Park, Florida, United States; 2 Department of Chemistry and Biochemistry, Millsaps College, Jackson, Mississippi, United States

## Abstract

Changes in lipid composition at membrane fusion sites in mating Tetrahymena are thought to involve the endoplasmic reticulum (ER), but its localization to these sites has not been observed. Here we show ER distribution during Tetrahymena mating using TtRET1-GFP and GFP-KDEL. We find that both markers localize to perinuclear membranes and tubular structures that connect perinuclear membrane to plasma membrane at fusion sites. Interestingly, both markers disappear from parental macronuclei after emergence of zygotic macronuclei. These similarities in localization of established ER marker, GFP-KDEL, and TtRET1-GFP reveal TtRET1-GFP as a useful new live cell marker for the ER in Tetrahymena.

**Figure 1. ER localization as reported by GFP-KDEL and TtRET1-GFP:**
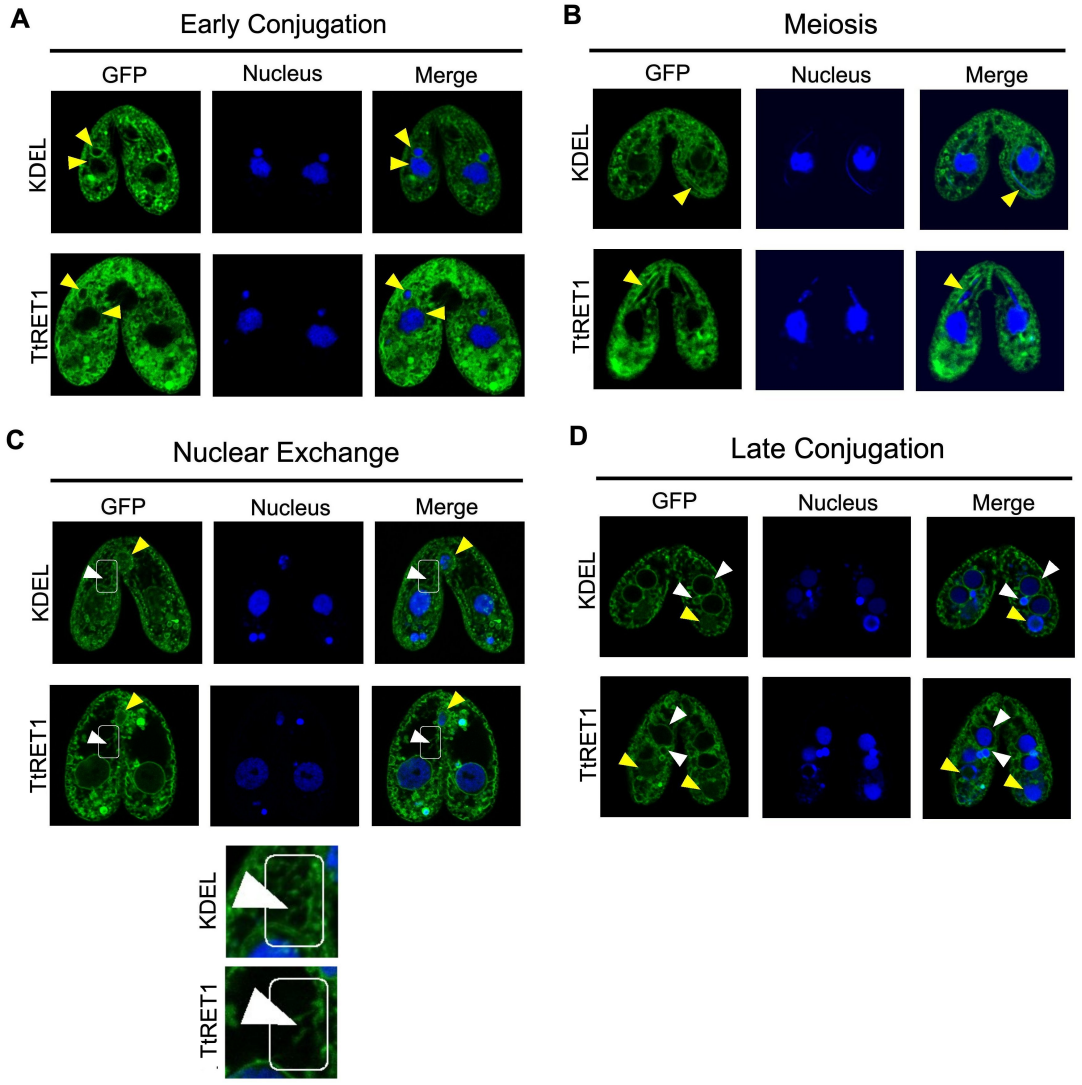
*Tetrahymena thermophila* of different mating types expressing either GFP-KDEL or TtRET1-GFP were starved then incubated together in a mating reaction (GFP-KDEL:WT; TtRET1-GFP:TtRET1-GFP). Mating reactions were treated with NucBlue (Thermofisher) to stain the nucleus then cells were subjected to fluorescence confocal microscopy. **A and B** . **KDEL and TtRET1 display perinuclear localization that extends throughout the cytoplasm.** Cells visualized during early conjugation (pre-meiosis) shown in A. Mating pairs visualized during meiosis shown in B. Yellow arrows indicate micro (small) and macronucleus (large) **C. KDEL and TtRET1 localize to tubular extensions, the conjugation junction, and exchanging micronuclei.** Cells visualized during nuclear exchange. Conjugation junction indicated by yellow arrowhead. White arrow indicated tubules and rectangle indicated region of tubulation. **D. KDEL and TtRET1 localization is lost from the parental macronucleus.** Cells visualized during late conjugation (after development of new macronuclei and micronuclei). Yellow arrows indicate parental macronuclei *.*

## Description

The plasma membrane is the boundary between the cell and its environment, and its functions depend in part on its lipid composition. This composition can be altered by numerous mechanisms, including lipid transfer from the endoplasmic reticulum (ER) (Lev, 2010; Wong et al., 2019) . Lipid transfer between the ER and plasma membrane occur at membrane contact sites (MCS) and these connections typically require expansion of the ER by ER tubulating proteins (Caldieri et al., 2017; Chang et al., 2017; Jozsef et al., 2014) . During Tetrahymena conjugation, the fusion between mating cells involves changes in the plasma membrane lipid composition (Kurczy et al., 2010) . Importantly, our previous work showed that the plasma membrane contacted membrane extensions from a membrane-bound compartment at the conjugation junction (sites of plasma membrane fusion during mating), but the identity of this compartment is unknown (Cole et al., 2015) . Given the role of the ER in regulating lipid composition we wondered if this membrane might be the ER.

To determine if the ER contacts the conjugation junction, we generated Tetrahymena cells in which we fluorescently tagged TtRET1 (TTHERM_00895610), the Tetrahymena homolog of mammalian reticulon (an ER tubulation protein) (Fuentes et al., 2023; Voeltz et al., 2006) , or GFP fused to an N-terminal signal sequence and C-terminal ER retention signal (target GFP to the ER lumen) (Xie et al., 2007) . We then used these cells in mating assays to observe ER localization during conjugation.

We found that throughout conjugation, both GFP-KDEL and TtRET1-GFP displayed perinuclear localization that extended into the cytoplasm ( Figure 1A -D). The perinuclear localization is maintained during meiosis as the localization takes on the crescent shape displayed by the micronucleus (yellow arrowheads in Figure 1B ) further supporting that KDEL and TtRET1 localize near the nucleus. It is important to note, that while only a single mating partner was expressing GFP-KDEL, both Tetrahymena displayed GFP localization suggesting that GFP-KDEL was transferred across the perforated membrane at the mating junction to the non-expressing mating partner. During nuclear exchange, both KDEL and TtRET1 appear to localize not only to the nuclear membranes but also to the conjugation junction (yellow arrowheads in Figure 1C ). Interestingly, we also observed GFP-KDEL and TtRET1-GFP localization to tubular structures that extend from the perinuclear area of the macronucleus towards the conjugation junction (white arrowheads and box; inset in Figure 1C ). This distribution of the ER is consistent with it transferring lipids to the plasma membrane during conjugation (Cole et al., 2015; Kurczy et al., 2010) . We also noticed that TtRET1-GFP showed increased localization to micronuclei that displayed condensed DNA and micronuclei near the posterior of the cell ( Figure 1C ). This suggests that TtRET1 could play a role in the degradation post-mitotic micronuclei (Liu & Yao, 2012) .

Lastly, during late conjugation we see prominent perinuclear localization of GFP-KDEL and TtRET1-GFP to the new micro and macronuclei (white arrowheads Figure 1D ) but virtually no localization to the old macronucleus which is destined for degradation during the conjugation process (yellow arrowheads in Figure 1D ). We also observed that GFP-KDEL and TtRET1-GFP localized to large circular structures that were not micronuclei ( Figure 1D ). Since this is a period of conjugation during which the old macronucleus is being degraded, we speculate that the circular structures may be autophagosomes (Akematsu et al., 2010, 2014; Liu & Yao, 2012) . The loss of TtRET1-GFP localization from the degrading parental macronucleus ( Figure 1D ) and its intense localization to post-mitotic micronuclei destined for elimination ( Figure 1C ) suggest that TtRET1 may play a role specifically in micronucleus degradation. While proteins such as ATG5 and ATG8 have been shown to regulate degradation of both micro and macronuclei (Bo et al., 2021; Liu & Yao, 2012) , deletion of VPS34, a kinase that is important for autophagosome formation, only leads to macronuclear degradation defects (Akematsu et al., 2014) . Moreover, unpublished data from our laboratory has shown that a Tetrahymena gene (TTHERM_00219300), which shows homology to SNX4, a gene known to regulate selective autophagy in saccharomyces cerevisiae (Nice et al., 2002) , localizes specifically to the degrading parental macronucleus but not micronuclei. This suggests that while there is commonality in the degradation of micro and macronucleus, there may also be distinct proteins that act in their respective degradation processes. Finally, unlike TtRET1-GFP, GFP-KDEL does not localize to micronuclei destined for elimination suggesting that while both markers label the ER, they may also label distinct structures. Future experiments will focus on co-expressing TtRET1 and KDEL to simultaneously look at their localization.

Taken together, our results show a distribution of the ER during conjugation that supports its potential role in lipid distribution throughout conjugation.

## Methods

**Generation of TtRET1-GFP Tetrahymena**

Monomeric enhanced GFP was fused to the C-terminus of TtRET1 gene (TTHERM_00895610) in the macronucleus via homologous recombination. First we amplified 1032bp long genomic region (containing the entire coding sequence without the stop codon) of TtRET1 using polymerase chain reaction then cloned into pmEGFP (gift from Turkewitz laboratory University of Chicago) using In-fusion cloning (Takara Bio). We then amplified and cloned TtRET1 3’UTR into pmEGFP-TtRET1 again via In-fusion. The resulting vector pmEGFP-TtRET1-mEGFP-3’UTR was linearized and introduced into Tetrahymena (both CU427 and B2086 cell lines) using biolistic transformation (Cassidy-Hanley et al., 1997) . Tetrahymena expressing endogenous TtRET1-GFP were selected by growth in SPP media and increasing concentrations of paromomycin.

**Tetrahymena mating reactions**

All incubations take place at 30 ^o^ C. TtRET1-GFP Tetrahymena (CU427 and B2086), GFP-KDEL (gift from Turkewitz laboratory University of Chicago) (CU428), and wild type (CU427) were grown overnight to 200,000 cells per ml. Cells were then washed and starved in 10mM Tris pH 7.4 overnight. After starvation TtRET1-GFP Tetrahymena were mixed at equal concentrations. Similarly, GFP-KDEL and wild type Tetrahymena were also mixed at equal concentrations. Mating reactions were then treated with NucBlue (Thermofisher) nuclear stain to assess stage of conjugation. Mating reactions were then immobilized using NiCL and imaged on Zeiss LSM 700 Confocal Laser Scanning Microscope, 63X oil with NA = 1.25 with Zen software.
